# ChatGPT for medical applications and urological science

**DOI:** 10.1590/S1677-5538.IBJU.2023.0112

**Published:** 2023-06-20

**Authors:** Leonardo O. Reis

**Affiliations:** 1 Universidade Estadual de Campinas Faculdade de Ciências Médicas Departamento de Urologia São Paulo Campinas Brasil UroScience e Departamento de Urologia, Faculdade de Ciências Médicas, Universidade Estadual de Campinas - UNICAMP, Campinas, São Paulo, Brasil; 2 Pontifícia Universidade Católica de Campinas Faculdade de Ciências da Vida Departamento de Imunoncologia São Paulo Campinas Brasil Departamento de Imunoncologia, Faculdade de Ciências da Vida, Pontifícia Universidade Católica de Campinas, PUC-Campinas, Campinas, São Paulo, Brasil

*To the editor,*

A Generative Pre-Trained Transformer (GPT) is an artificial intelligence (AI) algorithm designed to understand and generate human-like language. ChatGPT is a free and publicly available large language model developed by OpenAI. It uses advanced natural language processing algorithms, is trained on a vast corpus of data, and is not free of limitations and biases. AI is in its infancy and there is enough potential to be developed that will undoubtedly change our practices and human life. As an impactful tool, it can be used for the good and for the bad, and its responsible and moderated use is critical.

## What is chatGPT

A Generative Pre-Trained Transformer (GPT) is an artificial intelligence (AI) algorithm designed to understand and generate human-like language. ChatGPT is a free and publicly available large language model developed by OpenAI. It uses advanced natural language processing algorithms, trained on a vast corpus of data (1).

Training data includes a wide range of questions and prompts and a diverse set of texts, such as books, articles, and websites, allowing knowledge in many different domains. As an AI language model, its goal is to assist and provide useful responses to users who interact (2).

A PubMed search on March, 03, 2023 using the word “chatGPT” displayed 5 publications in 2022 and 72 in the first 2 months of the year of 2023 and another search on March, 09, 2023 retrieved additional 22 new documents. The studies range from literature reviews on the issue to clinical case reports that used chatGPT as a writing tool.

## ChatGPT formedical applications

ChatGPT has several potential applications in the field of medicine and healthcare. As an AI language model, ChatGPT has been trained on a vast amount of medical text data, including research papers, clinical reports, and electronic health records. This means that it has a deep knowledge base on medical topics and can generate text that may be instructive (3).

One potential application of ChatGPT in medicine is clinical decision support. By inputting patient data and symptoms, ChatGPT can generate recommendations for diagnosis and treatment based on the latest medical research and clinical guidelines. As any tool, when complementary to human expertise it can help improve the accuracy and efficiency of medical diagnosis and treatment, with potential to lead to better patient outcomes (4).

Another application of ChatGPT in medicine is natural language processing of electronic health records (EHRs). EHRs contain a vast amount of unstructured text data, and extracting meaningful information from these records can be time-consuming and challenging. ChatGPT can help automate this process by analyzing EHRs and identifying key information, such as patient diagnoses, treatments, and outcomes (5).

ChatGPT can also assist with patient education by generating easy-to-understand explanations of medical conditions and treatments. This can be particularly helpful for patients who may have difficulty understanding complex medical terminology or who may feel overwhelmed by the amount of medical information available online (6).

Overall, ChatGPT has several potential applications in medicine and healthcare, and its ability to generate text on a wide range of medical topics makes it a valuable tool for medical professionals, researchers, and patients alike. However, it's important to note that ChatGPT should be used as a complementary tool to human expertise and should not be relied upon as a substitute for professional medical advice or diagnosis.

## CHATGPT FOR MEDICAL WRITING

ChatGPT can be a valuable resource for medical writing in several ways. As an AI language model, ChatGPT has been trained on a vast amount of data, including medical literature, research articles, and clinical reports. This means that it has a vast knowledge base on medical topics and can generate text that is precise (7).

One way ChatGPT can help with medical writing is by providing assistance with grammar and syntax. Medical writing often involves complex terminology and jargon, and ChatGPT can help ensure that the language used in a medical document is grammatically correct and easy to understand (8).

ChatGPT can also help with writing medical reports, research papers, and other types of medical documents by providing suggestions for structure, formatting, and organization. For example, it can suggest appropriate headings and subheadings, provide examples of effective introductions and conclusions, and help ensure that the document flows logically and coherently (9).

Furthermore, ChatGPT can help with summarizing complex medical information in a way that is easy to understand for a lay audience. This can be particularly helpful for medical writers who are creating patient education materials or other types of health-related content (10).

Overall, ChatGPT's ability to generate grammatically correct text on a wide range of medical topics can be a valuable resource for medical writers looking to improve the quality and effectiveness of their work.

## ChatGPT for urological science

ChatGPT can be a useful resource for uro-logical science in several ways. As an AI language model, ChatGPT has been trained on a vast amount of text data, including scientific research papers, medical textbooks, and other authoritative sources on urological science. This means that it has a deep knowledge base on urological science (11).

One way ChatGPT can help with urological science is by providing assistance with writing research papers and clinical reports. Urological science involves complex medical terminology and jargon, and ChatGPT can help ensure that the language used in a research paper or clinical report is scientifically accurate (12).

ChatGPT can also help urological scientists with analyzing and interpreting data. By inputting data from urological studies or clinical trials, ChatGPT can generate text that provides insights into the findings, significance, and implications of the data. This can be particularly helpful for uro-logical scientists who need to communicate their research findings in a clear and concise way (13).

Furthermore, ChatGPT can assist with creating patient education materials on urological topics. Urological conditions can be complex and difficult to understand for patients, and ChatGPT can generate text that explains urological conditions and treatments in a clear and understandable way (14, 15). Overall, ChatGPT can be a valuable resource for urological scientists looking to improve the quality and effectiveness of their work.

## ChatGPT and references

ChatGPT is an AI language model that generates text based on patterns and statistical models learned from a large corpus of text data. While it can provide accurate and informative information, it does not have the ability to add exact references or citations to its writing.

However, as an AI language model, ChatGPT is trained on a vast amount of text data, including scientific research papers, academic journals, and other authoritative sources. This means that the information it provides is typically based on reliable and credible sources.

It's important to note that it is ultimately up to the user to ensure that the information is properly cited and referenced in any written work. If you are using ChatGPT to generate content for a research paper, article, or other type of written work, it's important to carefully review and fact-check the information it provides and to include proper citations and references to any sources used.

In summary, ChatGPT is a powerful tool that can provide information on a wide range of topics, but it is the user's responsibility to ensure that the information is properly cited and referenced in any written work.

## Ethics in the use of chatGPT

As an AI language model, ChatGPT has the potential to be a powerful tool for many different applications. However, there are important ethical considerations that must be taken into account in the use of ChatGPT (16).

First and foremost, the data that is used to train ChatGPT is critical. To ensure that ChatGPT is ethical and unbiased, the data that is used to train the model must be carefully selected and scrutinized. Training data that includes biased or discriminatory language can result in a model that perpetuates those biases and reinforces harmful stereotypes (17).

Another important ethical consideration is the potential misuse of ChatGPT. While ChatGPT can be used to provide helpful information and support to users, it can also be used to spread misinformation, hate speech, and other harmful content. It is important for developers and users of ChatGPT to be aware of this potential and take steps to mitigate it, such as implementing content moderation tools and educating users on responsible use.

There are also privacy and security concerns associated with the use of ChatGPT. Conversations with ChatGPT can contain sensitive personal information, and it is important for developers to take steps to protect this data from unauthorized access or use. Overall, ethics in the use of ChatGPT requires careful consideration of the data used to train the model, responsible use of the model to prevent harmful content, and safeguarding user privacy and security (18).

Researchers using AI tools should document this use in the methods or acknowledgements sections of the created manuscript, and AI tools cannot take any attribution of authorship, since it carries with responsibility and accountability for the work (19, 20).

AI is in its infancy, is not free of limitations and biases, and there is enough potential to be developed that will certainly change our practices and human life. As any impactful tool, it can be used for the good and for the bad, and its responsible and moderated use is key.

## ChatGPT limitations

Researchers are actively working to improve the capabilities and to address limitations that are common to most language models and AI systems in general.

While it is continually being updated with new data and fine-tuned by researchers and developers to improve its performance, the specific level of updating and the frequency of updates can vary depending on the resources and priorities of the team or organization responsible for maintaining ChatGPT. The current version was trained on a dataset that had a knowledge cutoff date of September 2021.

In addition to biases based on the sources of the data or the people who wrote it or programmers’ orientation, algorithmic bias may create systematic and repeatable errors with “unfair” outcomes in ways different from the intended algorithm.

Artificial intelligence hallucination may generate plausible sounding but incorrect or nonsensical answers that does not seem to be justified by its training data, such as claim to be human.

Limited access to the human commonsense knowledge, and inability to reason and learn and create like humans may generate struggle to understand the full context of a conversation or a piece of text, providing illogical, out of context, not relevant or not accurate responses (21).
